# MGFD: the maize gene families database

**DOI:** 10.1093/database/bax050

**Published:** 2017-06-26

**Authors:** Lei Sheng, Haiyang Jiang, Hanwei Yan, Xiaoyu Li, Yongxiang Lin, Hui Ye, Beijiu Cheng

**Affiliations:** Key Laboratory of Crop Biology of Anhui Province, Anhui Agricultural University, Hefei 230036, China

Due to circumstances beyond the journal's control, the database for the above article is no longer accessible. Apologies for the inconvenience.

